# Corneal Sensitivity and Dry Eye Symptoms in Patients with Keratoconus

**DOI:** 10.1371/journal.pone.0141621

**Published:** 2015-10-23

**Authors:** Lóránt Dienes, Huba J. Kiss, Kristóf Perényi, Zoltán Z. Nagy, M. Carmen Acosta, Juana Gallar, Illés Kovács

**Affiliations:** 1 Semmelweis University, Department of Ophthalmology, Budapest, Hungary; 2 Instituto de Neurociencias, Universidad Miguel Hernandez-CSIC, San Juan de Alicante, Spain; University of Oklahoma Health Sciences Center, UNITED STATES

## Abstract

**Purpose:**

To investigate corneal sensitivity to selective mechanical, chemical, and thermal stimulation and to evaluate their relation to dry eye symptoms in patients with keratoconus.

**Methods:**

Corneal sensitivity to mechanical, chemical, and thermal thresholds were determined using a gas esthesiometer in 19 patients with keratoconus (KC group) and in 20 age-matched healthy subjects (control group). Tear film dynamics was assessed by Schirmer I test and by the non-invasive tear film breakup time (NI-BUT). All eyes were examined with a rotating Scheimpflug camera to assess keratoconus severity.

**Results:**

KC patients had significatly decreased tear secretion and significantly higher ocular surface disease index (OSDI) scores compared to controls (5.3±2.2 vs. 13.2±2.0 mm and 26.8±15.8 vs. 8.1±2.3; p<0.001). There was no significant difference in NI-BUT between the two groups (KC: 9.8±4.8 vs. control: 10.7±3.8; p>0.05). The mean threshold for selective mechanical (KC: 139.2±25.8 vs. control: 109.1±24.0 ml/min), chemical (KC: 39.4±3.9 vs. control: 35.2±1.9%CO_2_), heat (KC: 0.91±0.32 vs. control: 0.54±0.26 Δ°C) and cold (KC: 1.28±0.27 vs. control: 0.98±0.25 Δ°C) stimulation in the KC patients were significantly higher than in the control subjects (p<0.001, for all parameters). No correlation was found between age and mechanical, chemical, heat or cold thresholds in the patients with KC (p>0.05), whereas in the control subjects both mechanical (r = 0.52, p = 0.02), chemical (r = 0.47, p = 0.04), heat (r = 0.26, p = 0.04) and cold threshold (r = 0.40, p = 0.03) increased with age. In the KC group, neither corneal thickness nor tear flow, NI-BUT or OSDI correlated significantly with mechanical, chemical, heat or cold thresholds (p>0.05 for all variables).

**Conclusions:**

Corneal sensitivity to different types of stimuli is decreased in patients with keratoconus independently of age and disease severity. The reduction of the sensory input from corneal nerves may contribute to the onset of unpleasant sensations in these patients and might lead to the impaired tear film dynamics.

## Introduction

Keratoconus is a bilateral, non-inflammatory, progressive disorder characterized by corneal thinning and protrusion that leads to corneal surface distortion. Although the exact pathogenesis of keratoconus remains poorly understood, several genetic, biochemical, and biomechanical factors have been implicated in the development of this ectatic disease [1,2]. The involvement of corneal nerves in the pathogenesis of keratoconus has not yet been investigated, and only the prominence and visibility of central corneal nerves have been reported as a characteristic sign of keratoconus [3]. Reduced density and abnormal morphology of the corneal subbasal and stromal nerves has been described in patients with keratoconus using confocal corneal miscroscopy [4–12] as well as with histological examinations [13,14]. Whether these morphological changes are primary or secondary pathologic manifestations is currently not known.

Normal blinking and tearing reflexes are controlled by a reflex arc that includes the ocular surface, intact corneal innervation, and lacrimal glands. Compromised function in any part of this reflex arch results in impaired tear secretion and ocular surface health [15]. The precorneal tear film protects the ocular surface from external damage but corneal nerve endings are exceedingly close to the surface and can easily react to different types of environmental stimuli. In addition to the sensory and protective functions, corneal nerves also have trophic effects on the cornea by secreting neuropeptides, neurotrophins, and growth factors, and thus are involved in the maintenance of epithelial integrity, the modulation of cell proliferation, and in maintaining normal corneal structure and function [16–19].

Although there are several reports on the significantly lower mechanical sensitivity in keratoconus patients using a Cochet-Bonnet esthesiometer [20–24], the level of impact on the various functional types of corneal sensory nerve fibers during keratoconus has not been established in detail. Moreover, there is no available data on the contribution of the different types of corneal sensory nerve endings to the sensory impairment and to impaired tear secretion in keratoconus patients. The evaluation of the relationship between sensory and tear film impairment might help to understand the pathophysiology of corneal nerve loss in this population. The aim of this study was to investigate corneal sensitivity to selective mechanical, chemical, and thermal stimulation and to evaluate its relation to keratoconus severity and to dry eye symptoms in patients with keratoconus.

## Materials and Methods

The original version of the Belmonte noncontact gas esthesiometer was used to explore corneal sensitivity thresholds to selective mechanical, chemical, heat, and cold stimuli [25–27] in one randomized eye in 19 patients (17–47 years) with bilateral mild or moderate keratoconus (KC group) and in 20 healthy refractive surgery candidates (control group) (21–55 years) of both sexes. Sample size was based on a power calculation (power 0.90; p = 0.05) using SDs obtained in the previous studies from our institution. Eyes with severe keratoconus were excluded from the study because potential stromal haze or scar formation can alter Scheimpflug image acquisition and corneal sensitivity measurements. The diagnosis of keratoconus was based on classic corneal biomicroscopic and topographic findings in accordance with the criteria of Rabinowitz et al [28]. Patients with keratoconus were asked whether they rubbed their eyes or experienced previous ocular trauma. Inclusion criteria for the control group included a refractive error less than 5.00 diopters (D) sphere and astigmatism less than 3.00 D and no family history of keratoconus. Participants in the control group did not have any clinical signs and/or symptoms of dry eye (ocular surface disease index—OSDI score <10) or significant ocular surface disease and were not using eye drops. Subjects with ophthalmic conditions other than keratoconus including blepharitis, meibomitis, lid abnormalities as well as contact lens wearers were also excluded. Both eyes of each patient had a complete ophthalmologic evaluation including slitlamp biomicroscopy, ophthalmoscopy, Scheimpflug imaging and assessment of tear flow and non-invasive tear film breakup time. Subjects who showed significant corneal staining (>Grade 2, Oxford Scale) [29] were excluded because corneal epitheliopathy could potentially be a confounding factor affecting the ocular surface sensory responses [18,30,31]. All patients completed a questionnaire to assess dry-eye disease symptoms (ocular surface disease index—OSDI). None of the subjects received any drops at least 6 hours before the measurements. Tear film dynamics was assessed by the Schirmer I test without anesthesia and by measuring the non-invasive tear film breakup time with a specific instrument (Keeler Tearscope Plus, Keeler, Windsor, UK). The study was conducted in compliance with the Declaration of Helsinki, applicable national and local requirements regarding the ethics committee and institutional review boards. Ethical approval was obtained from the Institutional Review Board (Semmelweis University Regional and Institutional Committee of Sciences and Research Ethics). A written informed consent was obtained before the examination from each patient or from the parent on behalf of the minors/children.

### Scheimpflug assessment

All eyes were examined with a rotating Scheimpflug camera (Pentacam HR, Oculus Optikgerate, Wetzlar, Germany), used by three trained examiners without application of dilating or anesthetic eye drops or previous tonometry. The readings were taken as recommended in the instruction manual. For local posterior elevation measurements, the reference surface was set to best fit sphere (BFS) with fixed 8- mm-diameter settings. Keratometry at the steep (Ks) and flat (Kf) meridians and corneal thickness at the thinnest point (ThCT) were measured in both eyes.

### Measuring non-invasive tear film breakup time

The non-invasive tear film breakup time (NI-BUT) was measured using the Keeler Tearscope Plus immediately after a complete blink. The Keeler Tearscope Plus was attached to a slit lamp (Topcon SL-D2, Topcon Medical Systems, Oakland, NJ, USA) in a fixed position to obtain a full coverage of the cornea. The measurement of non-invasive tear film breakup time with Tearscope Plus is based on the projection of a cylindrical source of cool white fluorescent light onto the cornea so that tear film breakup could be observed at any point over the corneal surface. The tear film was recorded by a digital camera (Topcon DV-3, Topcon Medical Systems, Oakland, NJ, USA) attached to the slit lamp, captured videos were exported at a spatial resolution of 1024 × 768 pixels and were analyzed by a masked observer. The non-invasive tear film breakup time was defined as the time from the last blink when visible deterioration of the projected rings was detectable during the continuous recording. In each subject, NI-BUT was averaged from three consecutive measurements.

### Corneal esthesiometry

Mechanical, chemical, and thermal (hot and cold) thresholds were determined at the center of the cornea using a Belmonte's gas esthesiometer. The Belmonte non-contact esthesiometer allows exploration of different types of sensory fibers, such as mechanosensory fibers that respond to mechanical forces; polymodal nociceptive fibers that respond to mechanical forces, irritants, extreme temperatures, and endogenous inflammatory mediators; and cold fibers that are activated mainly by the decrease of temperature [26]. It is known that during mechanical stimulation, when air at increasing flow rates is applied to the corneal surface at a temperature of 34°C, the corneal polymodal nociceptors and mechanoreceptors are predominantly activated. With gas mixtures of increasing CO_2_ concentration, a proportional decrease in pH occurs at the corneal surface acting as a specific stimulus for polymodal nociceptors of the cornea with an intensity proportional to the local pH reduction [32]. Likewise, hot air applied to the cornea selectively activates polymodal nociceptors, simultaneously silencing the spontaneously active cold receptors. Finally, moderate cooling exclusively stimulates cold receptors, whereas polymodal nociceptors appear to be weakly recruited by cold air only with corneal temperatures below 29°C [26]. A specific instrument with a rotary potentiometer was built to record intensity rating immediately after stimulation. Subjects were instructed to adjust the potentiometer to the corresponding intensity of the sensations arising during stimulation. A specific computer software written in MatLab program (The MathWorks, Natick, MA) was used to sample the data acquired from the potentiometer and to convert it to numeric values on a 10 unit scale. We measured with the potentiometer the intensity of the irritation sensation evoked by selective mechanical, chemical, and thermal stimuli applied on the central cornea of participants using the gas esthesiometer. Mechanical, chemical (CO_2_ in air), and cold stimuli were used during three-second air pulses of adjustable flow rate, composition (CO_2_%) and temperature. Mechanical thresholds were determined by using the method of levels as described previously elsewhere [25]. Mechanical stimulation consisted of variable flows of filtered medicinal air (50 to 200 ml/min). Air was heated at the tip of the probe at 50°C so that it reached the ocular surface at 34°C to prevent a change in corneal temperature caused by the airflow [25]. Thermal stimulation was done by cooling or heating the air to produce the required changes in basal corneal temperature (from -3°C to +3°C) with a flow 10 ml/min below mechanical threshold. For chemical stimulation, a mixture of medicinal air with different concentrations of CO_2_ (30 to 50%) was used at 50°C at the tip of the probe and with a flow rate of 10 ml/min below mechanical threshold. After corneal esthesiometry, the Schirmer test was performed.

### Statistical analysis

Statistical analysis was performed with SPSS software (version 21.0, IBM Inc., Chicago, IL, USA). The Shapiro-Wilk W test was used to assess normal distribution of the variables. Due to non-normality of data the Mann–Whitney U test was used for group comparisons. Spearman correlation analysis was used to determine the correlation between corneal sensitivity and age or pachymetric severity of keratoconus. In all analyses a p value less than 0.05 was considered as statistically significant.

## Results

There was no significant difference in age and gender between the keratoconus and the control group (p>0.05, Table 1). Patients with keratoconus had signifiancantly higher steep and flat keratometry values and significantly lower thinnest corneal thickness compared to normals (Table 1). Patients with keratoconus had significatly decreased tear secretion and significantly higher OSDI scores compared to controls (p<0.001, Table 1). There was no significant difference in tear film breakup time between the two groups (p>0.05, Table 1).

**Table 1:** Demographic, topographic and tear film characteristics of the control and the keratoconus groups.

	*Control*	*Keratoconus*	*P*
***Age (years)***	30.2 ± 5.3	28.9 ± 6.3	0.55
***Gender (male/female)***	12 / 8	10 / 9	0.89
***Keratometry steep axis (D)***	43.9 ± 1.5	49.0 ± 5.7	<0.001
***Keratometry flat axis (D)***	43.1 ± 1.4	45.8 ± 5.3	<0.001
***Thinnest corneal thickness (μm)***	551.7 ± 13.9	422.4 ± 77.9	<0.001
***Schirmer I test***	13.2 ± 2.0	5.3 ± 2.2	<0.001
***NI-BUT (s)***	10.7 ± 3.8	9.8 ± 4.8	0.31
***OSDI score***	8.1 ± 2.3	26.8 ± 15.8	<0.001

Data are mean ± SD values in the control (n = 20) and in the keratoconus groups (n = 19). Note: P: Mann–Whitney U test.

**Fig 1:**
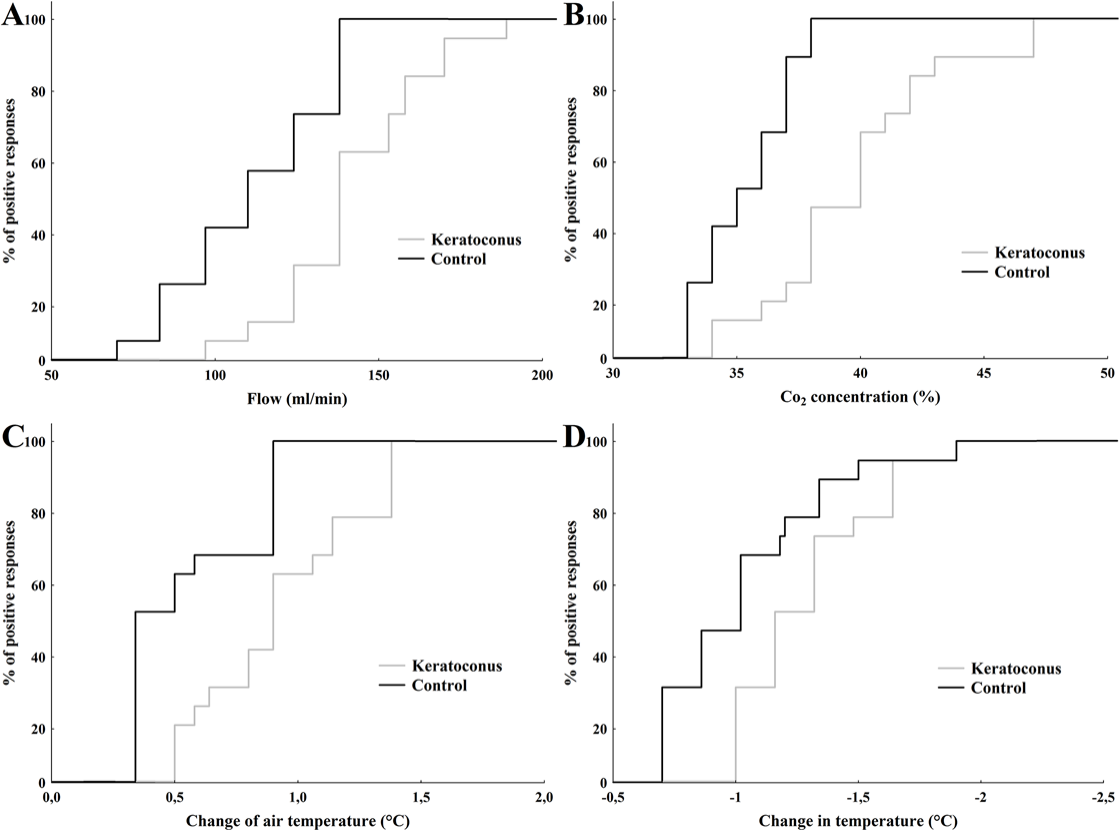
Cumulative distribution of sensation thresholds to selective stimulation of the central cornea in control subjects and keratoconus patients. (A) air pulses of increasing flow (mechanical stimulation), (B) pulses with increasing CO_2_ concentration (chemical stimulation), (C) pulses of air at increasing temperatures (hot thermal stimulation), and (D) pulses of air at decreasing temperatures (cold thermal stimulation), in KC patients (gray line) and controls (black line).

**Fig 2:**
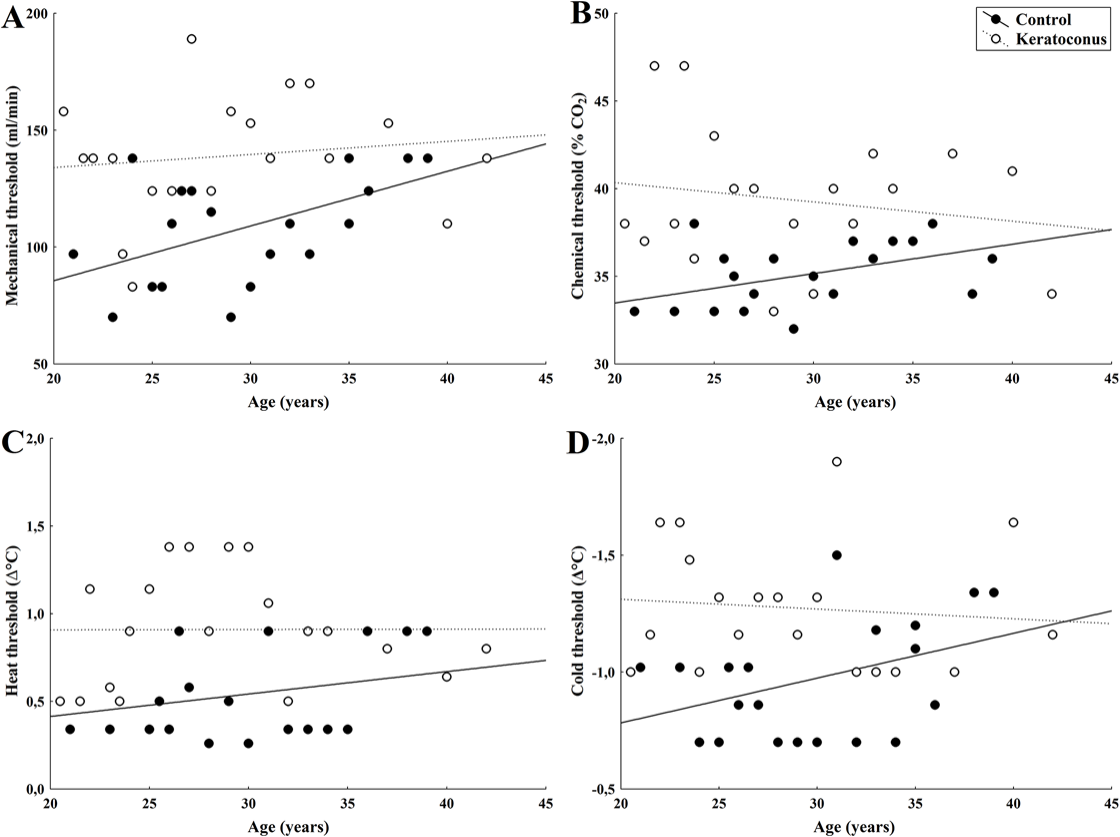
Relationship between age and corneal sensitivity threshold to mechanical (A), chemical (B), heat (C), and cold (D) stimulation in KC patients and in control subjects. Regression lines (solid: control; dotted: KC) are also plotted (see text for details on R coefficients and P-values).

The threshold sensitivity to mechanical stimulation with air pulses of neutral temperature applied to the center of the cornea in the patients with KC was significantly higher than those observed in the control subjects (p<0.001; Table 2, Fig 1A). No correlation was found between mechanical threshold and age in the patients with KC (r = 0.13, p = 0.58; Fig 2A), whereas in the control subjects, mechanical threshold increased proportionally with age (r = 0.52, p = 0.02; Fig 2A).

**Table 2:** Sensation thresholds to selective stimulation of the cornea.

*Stimulation*	*Control*	*Keratoconus*	*P*
***Mechanical (ml/min)***	109.1 ± 24.0	139.2 ± 25.8	<0.001
***Chemical (%CO***_***2***_ ***)***	35.2 ± 1.9	39.4 ± 3.9	<0.001
***Heat (Δ°C)***	0.54 ± 0.26	0.91 ± 0.32	<0.001
***Cold (Δ°C)***	0.98 ± 0.25	1.28 ± 0.27	0.001

Data are mean ± SD values in the control (n = 20) and in the keratoconus groups (n = 19). Note: P: Mann–Whitney U test.

The mean sensation threshold for selective chemical stimulation was significantly higher in patients with KC than in the control group (p<0.001; Table 2, Fig 1B). Chemical thresholds did not tend to increase with age in the subjects with KC (r = -0.17, p = 0.46; Fig 2B), contrary to the responses of the control subjects (r = 0.47, p = 0.04; Fig 2B).

A significantly higher threshold value was obtained with heat stimulation in patients with KC than in the control group (p<0.001; Table 2, Fig 1C), with no correlation between threshold and age (r = 0.01, p = 0.98; Fig 2C) contrary to the responses of the control subjects, in whom threshold and age correlated positively (r = 0.26, p = 0.04; Fig 2C).

Similarly, an elevated threshold value to cold stimulation was observed in patients with KC compared to the control individuals (p = 0.001; Table 2, Fig 1D). Cold threshold responses did not correlate with age in patients with KC (r = -0.09, p = 0.69; Fig 2D), whereas in control subjects the correlation was significant (r = 0.40, p = 0.03; Fig 2D).

In the keratoconus group, corneal thickness did not correlated significantly with threshold values of mechanical, chemical, heat or cold stimulation (p>0.05 for all variables, S1 Fig). Similarly, threshold values of mechanical, chemical, heat or cold stimulation did not correlated to tear flow (p>0.05 for all variables, S2 Fig), NI-BUT (p>0.05 for all variables, S3 Fig) or OSDI score (p>0.05 for all variables, S4 Fig). In the keratoconus group, there was no correlation between thinnest corneal thickness and tear flow, NI-BUT or OSDI values (p>0.05 for all variables).

## Discussion

The pathophysiology of keratoconus has not yet been completely elucidated, though there appear to be some environmental and genetically predisposing factors in its development [2]. In previous studies using *in vivo* confocal microscopy, subbasal nerve density has been shown to be lower in corneas with keratoconus and appeared more tortuous in these corneas as compared to controls, with abnormal architecture affecting primarily the region of the cone [7–12,23]. It has also been demonstrated, that the decrease in nerve density is significantly correlated with the loss of corneal sensitivity to contact mechanical stimulation, this correlation being stronger in patients who wore contact lenses [6,21]. Although there are also some reports on impaired tear secretion in patients with keratoconus [33,34], the relationship between abnormal ocular surface innervation and tear film dynamics remains unclear.

In this study we have demonstrated that in keratoconus patients both corneal sensitivity and tear secretion are reduced. Our results show a significantly increased threshold for conscious detection of mechanical, chemical and thermal stimuli applied to the cornea in patients with keratoconus, in comparison with age-matched control subjects. Within the keratoconus group, patients showed the same profile of sensitivity deficiency irrespective of their age, disease severity and tear function, suggesting that sensory deterioration appears early in the development of keratoconus and is independent of age or ocular surface wetness. Apart from corneal sensitivity threshold values, neither tear secretion, nor unpleasant sensations correlated with keratoconus severity or age demonstrating that in the case of keratoconus corneal hypesthesia with profound abnormality in sensory input and abnormal tear secretion develops early in the disease and remains unaltered independently of age.

Our finding, that changes in tear flow and tear film breakup time are not related to disease severity or patient’s age is in good harmony with previous reports, where lack of correlation was described between topographic severity of keratoconus and dry eye symptoms or tear film parameters [34]. The significantly reduced corneal sensitivity to mechanical stimulation measured with the Cochet-Bonnet esthesiometer has already been described in keratoconus patients, however this device has limited accuracy and only stimulates mechanosensory nerve fibers. Hence, in the present study using the Belmonte’s gas esthesiometer we have shown for the first time, that corneal sensory nerve impairment in keratoconus affects all types of corneal sensory nerve endings. The importance of this finding is, that not only sensory nerve input that is responsible for reflex tear secretion (that is, the activity of polymodal nociceptors) but those responsible for maintaining basal tear secretion (that is, the activity of corneal cold thermoreceptors) are also considerably involved in corneal sensitivity loss in KC patients. It has already been shown, that the stimulation of corneal polymodal and mechano- nociceptor fibers results in unpleasant feeling and reflex tearing [17], while the spontaneous activity of corneal cold sensitive nerve fibers is responsible for maintaining basal tear secretion [35]. Cold thermoreceptors are able to detect slight (< 0.5°C) variations in ocular surface temperature and also changes in tear film osmolarity [36], such as those occurring during tear film evaporation, and thus regulating tear flow. Under normal circumstances, the continuous impulse firing from cold thermoreceptors represents a tonic stimulus for basal tear fluid secretion, conceivably activating the lacrimal glands and goblet cells through the parasympathetic fibers from the superior salivary nucleus. During the interblink period, ocular surface temperature falls gradually from approximately 34°C at a rate of 0.3°C/s due to tear film evaporation [37]. Corneal cold receptor endings exhibit a remarkably high sensitivity for dynamic temperature reductions and are thus able to encode into their background firing frequency such small temperature oscillations [38]. In keratoconus patients in whom basal tear secretion is reduced, the lower number of cold fibers that remain functional presumably fire at higher frequency and evoke dryness sensations even though their summated sensory inflow may be still insufficient to maintain the fraction of the tear flow dependent on cold fiber tonic effects on parasympathetic pathways.

In this study we also have demonstrated, that in comparison to healthy controls, in keratoconus patients lower tear secretion and tear film breakup time are associated with the presence of unpleasant ocular surface sensations. Presumably, the altered excitability of corneal cold receptors is the origin of the lowered sensitivity and dry eye sensations and other disaesthesias reported by the patients with keratoconus as the origin of unpleasant sensations in ocular surface dryness is mainly attributed to the abnormal activity of cold receptors secondary to ocular surface desiccation and tear film hyperosmolarity [36,38]. However, there is a complex relationship between ocular surface sensory function and tear film production, and the lack of correlation between subjective symptoms, tear rate reduction (as measured by the Schirmer test), and ocular surface damage (evaluated with fluorescein and Lissamine green staining) is well known [39]. It has been proposed previously, that changes in the activity of corneal sensory nerves, which are part of the lacrimal functional unit, modify tear secretion and may lead to ocular dryness [15,40,41]. In the case of keratoconus, it is possible that structural changes of the cornea causes an impairment of sensory nerve activity and a reduction of corneal sensitivity, and as a consequence of their reduced sensory input, tear secretion driven by tonic nerve activity is decreased, thus causing ocular symptoms. Our results demonstrate that there is a significantly decreased tear flow in keratoconus patients with the impairment of both cold- and mechanoreceptor function, and thus both basal and reflex tearing are altered. Taken together these findings it appears reasonable to conclude that in patients with keratoconus the reduced reflex tear secretion is caused by the reduced input to the brain from corneal mechanical and polymodal receptors while the reduction in basal tear secretion is the result of the decreased input from corneal cold receptors secondary to their morphological and functional impairment. The reduced sensory input could be the result of the reduced nerve density [4–12] and/or produced by the reduction of the excitability of sensory nerve endings due to an altered expression of ion channels in trigeminal sensory neurons. However, from our results, it cannot be determined whether this is a direct effect of the disease on sensory nerve endings, or is secondary to the ocular surface desiccation, as is the case in patients with dry eye of other origins [42,43]. In keratoconus, the accelerated apoptosis and lysis of basal epithelial cells with the release of intracellular proteolytic enzymes might be the key triggers of subsequent destructive events involving the underlying corneal tissue, including the nerve endings [44]. It has also been shown that the destructive process in keratoconus involves not only the nerves but their associated Schwann cells also, which also express proteolytic enzymes [14]. Although this hypothesis could explain the reduction of nerve density frequently reported using *in vivo* confocal microscopy investigations, no clear evidence is available to explain why these nerves are becoming thicker and showing abnormal morphologic features. It has been shown that sensory nerve fibers are extremely sensitive to signals released after injury, resulting in transient nerve sprouting at the site of injury and those sprouting terminals could play a stimulatory role in the healing process by releasing neuropeptides and other factors at the wound site [45,46]. The role of neuropeptides such as nerve growth factor (NGF) in controlling this nerve overgrowth was described in experimental skin injury [47] and is supported by the clinical evidence of keratoconus progression after injury-induced fifth nerve palsy [48]. An altered expression of other growth factors, and cytokines such as interleukin 1 and 6, intercellular and vascular cell adhesion molecules has also been reported in keratoconus [49–54]. These substances are well known for their neurotrophic effects and may have a role in the pathophysiologic features and subsequent rearrangements and functional changes of corneal nerves in keratoconus. Whether the abnormal sensory input as a result of impaired function of corneal nerve endings might have a role in the development of abnormal ocular surface sensations and thus evoking eye rubbing is yet unclear but these processes might contribute to the progression of keratoconus. However, the relationship of the corneal nerve deterioration and the progressive corneal thinning in keratoconus needs to be elucidated and further studies are recommended as relationship would be better described when longitudinal data of patients with the entire spectrum of the disease were analyzed. Our future analyses aim to examine whether changes in corneal sensory function precedes corneal thinning or whether early signs of corneal ectasia could be detected before sensory nerve impairment. One limitation of our study was that the measurements were made on subjects representing the characteristics of our clinic population. Further studies are recommended as the difference between keratoconus patients and healthy subjects would be better described when data from larger pools and other populations were analyzed.

As a summary, in this study we have demonstrated that corneal sensitivity to different types of stimuli is decreased in patients with keratoconus. The significantly impaired sensitivity suggests that axonal damage and/or altered expression of membrane ion channels involved in transduction and membrane excitability evenly affects the different types of corneal nerve terminals. Our finding that changes in corneal sensitivity and tear flow are not related to disease severity or patient’s age suggests that there is an early development of impaired corneal nerve function in keratoconus. Although the exact mechanism of corneal nerve damage in keratoconus is still unknown, these structural and neural changes may play a role in the impaired tear secretion as well as in the abnormal ocular sensations experienced by keratoconus patients. Our results highlight the need for further studies on the impact of impaired tear secretion and sensory nerve function on anatomical and visual results following corneal collagen cross linking therapy or keratoplasty in eyes with keratoconus. Better understanding of the pathophysiology of the impaired ocular sensitivity in keratoconus may also help to elucidate the role of damaged sensory nerve terminals in progressive corneal thinning, and thus, might help in timing corneal collagen cross linking therapy to prevent further stromal thinning.

## Supporting Information

S1 Fig Relationship between corneal thickness and corneal sensitivity threshold to mechanical (A), chemical (B), heat (C), and cold (D) stimulation in patients with keratoconus. (TIF)

S2 Fig Relationship between Schirmer’s test and corneal sensitivity threshold to mechanical (A), chemical (B), heat (C), and cold (D) stimulation in patients with keratoconus. (TIF)

S3 Fig Relationship between tear film breakup time and corneal sensitivity threshold to mechanical (A), chemical (B), heat (C), and cold (D) stimulation in patients with keratoconus. (TIF)

S4 Fig Relationship between OSDI score and corneal sensitivity threshold to mechanical (A), chemical (B), heat (C), and cold (D) stimulation in patients with keratoconus. (TIF)
