# Supplementary figures and images for: Corneal Sensitivity and Dry Eye Symptoms in Patients with Keratoconus
Source: PLoS One. 2015 Oct 23;10(10):e0141621. doi: 10.1371/journal.pone.0141621 (PMC4619831; doi:10.1371/journal.pone.0141621)

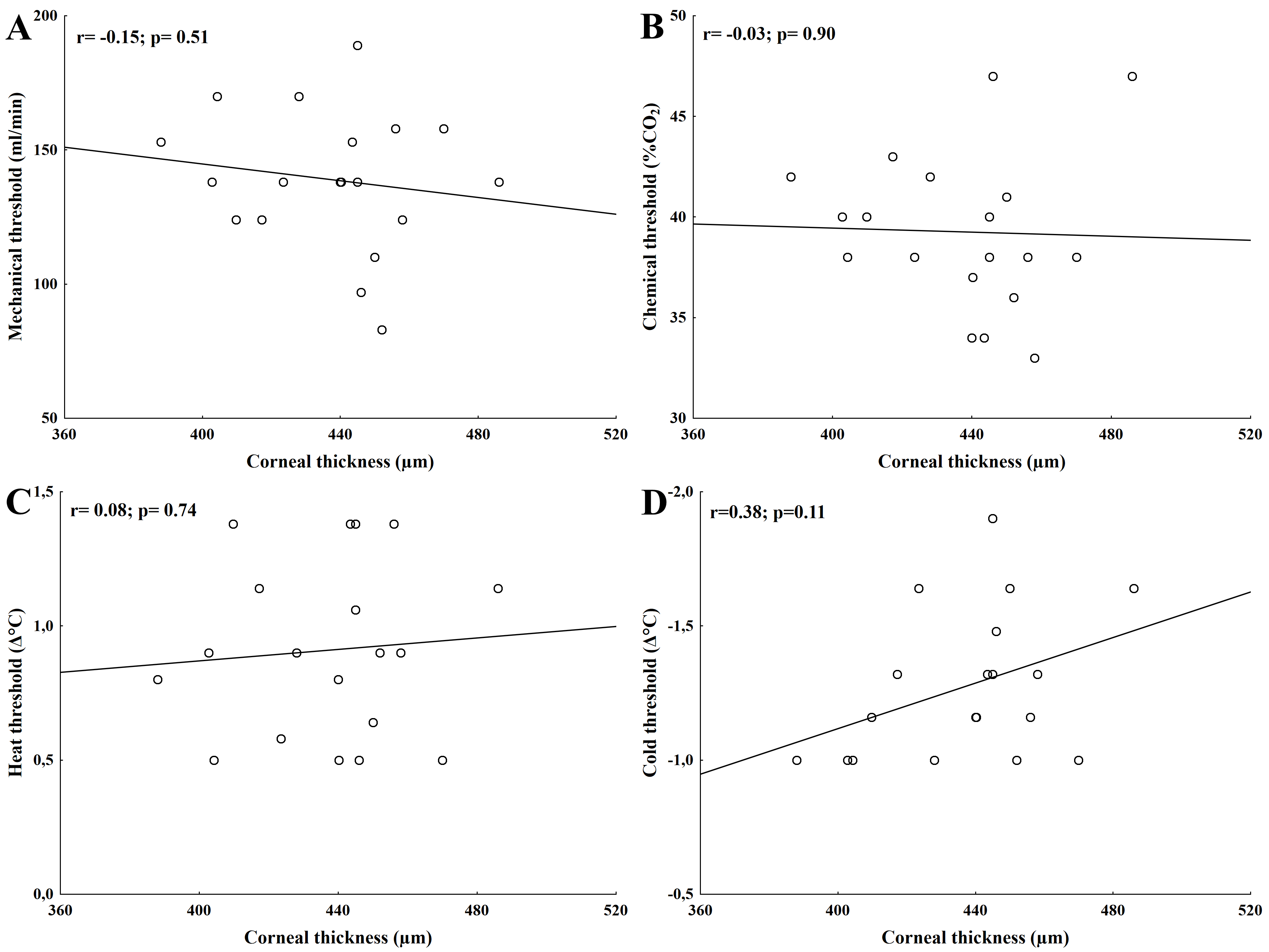

Supplement: S1 Fig — (TIF) [file pone.0141621.s001.tif]

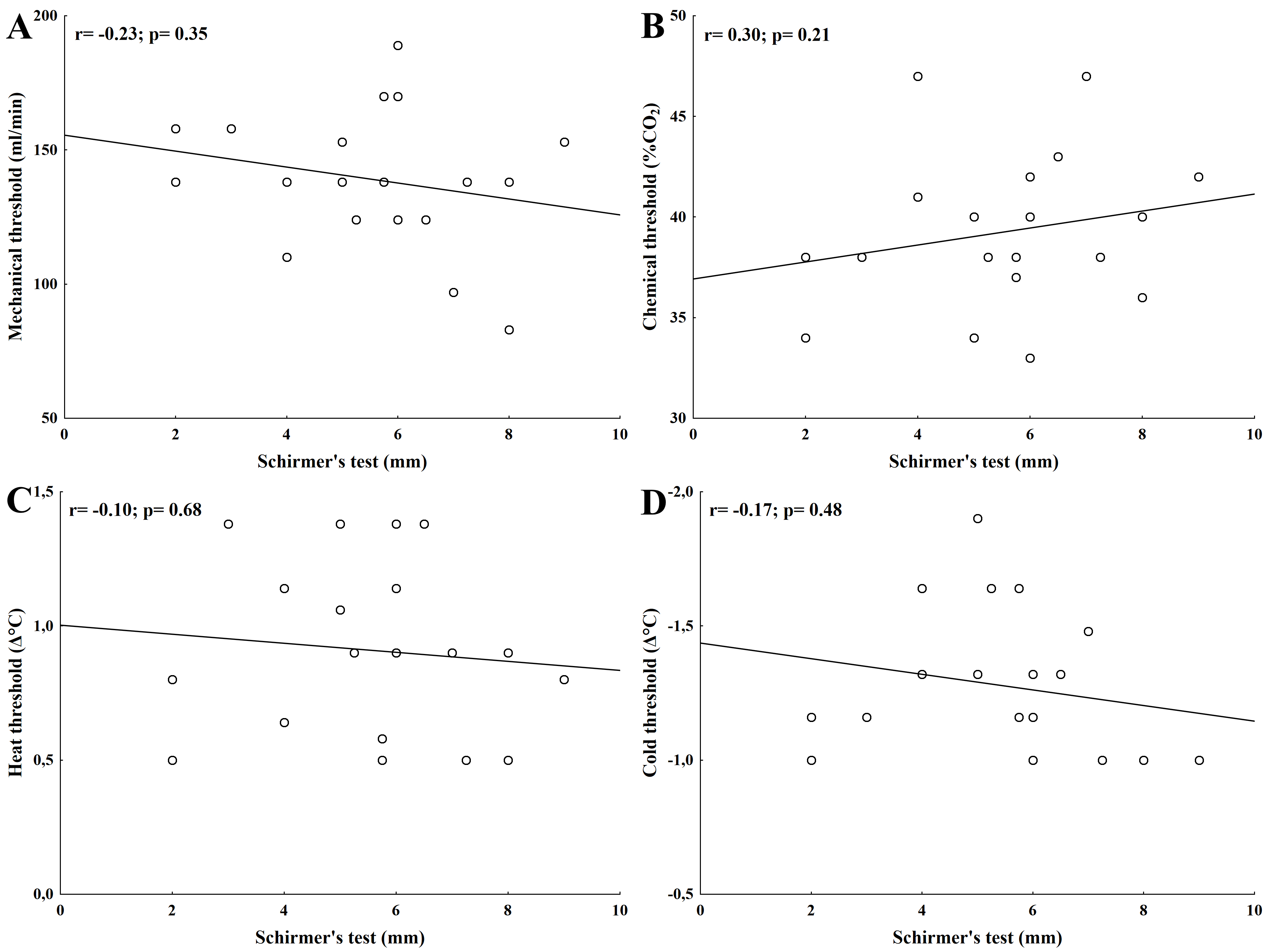

Supplement: S2 Fig — (TIF) [file pone.0141621.s002.tif]

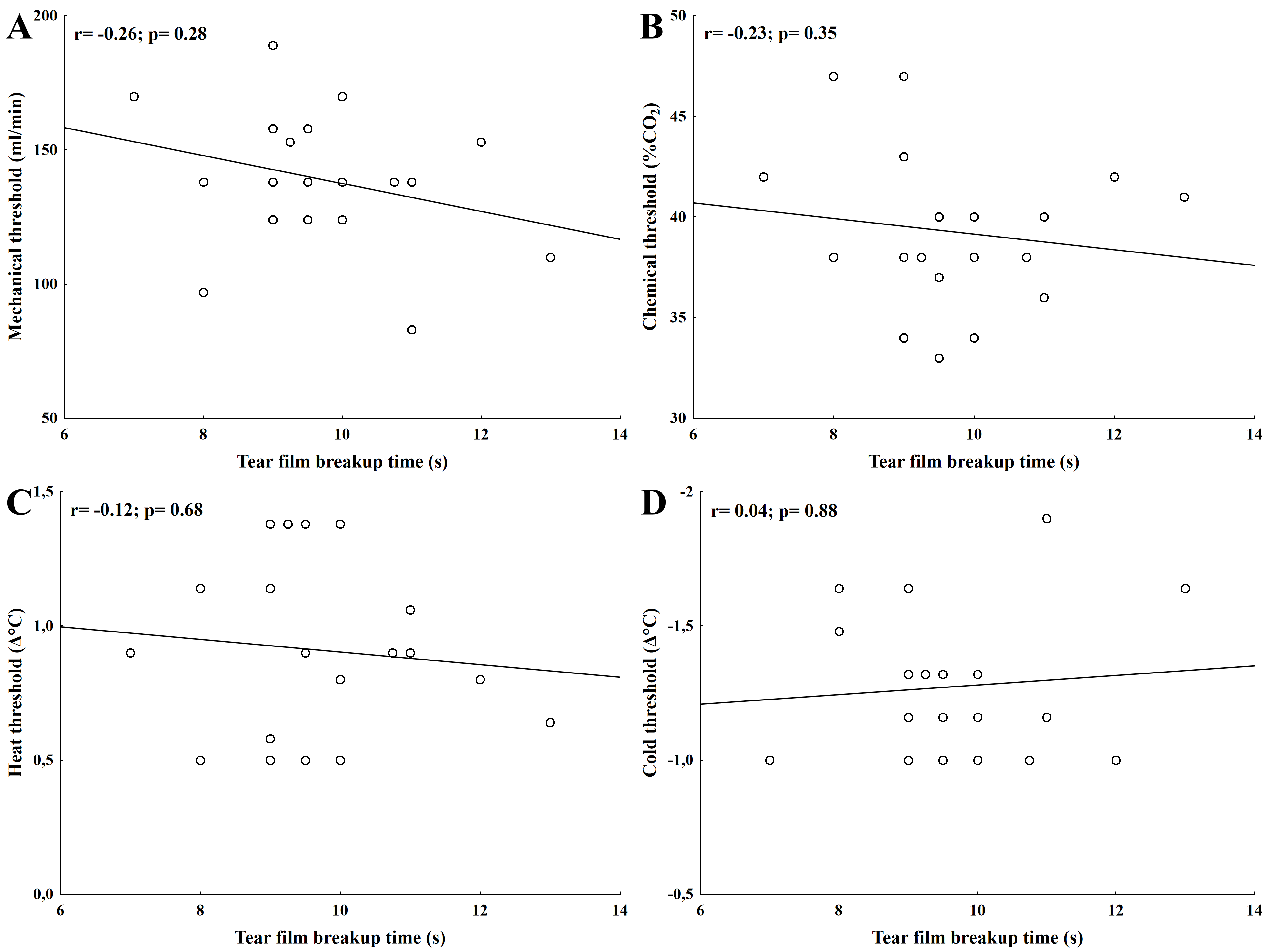

Supplement: S3 Fig — (TIF) [file pone.0141621.s003.tif]

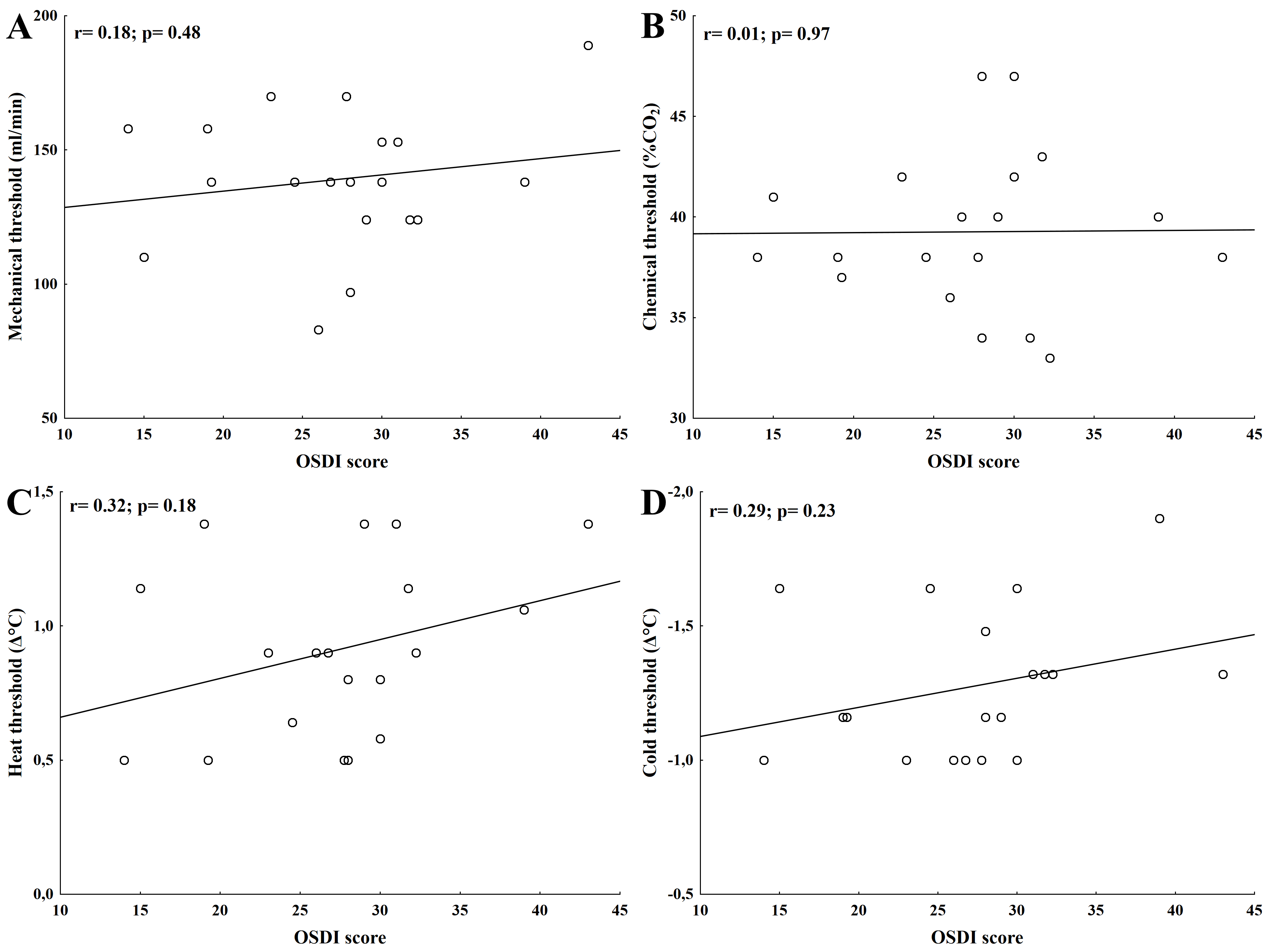

Supplement: S4 Fig — (TIF) [file pone.0141621.s004.tif]
